# Effect of Infarct Location and Size on Left Atrial Function: A Cardiovascular Magnetic Resonance Feature Tracking Study

**DOI:** 10.3390/jcm11236938

**Published:** 2022-11-24

**Authors:** He Zhang, Zhaoxin Tian, Huaibi Huo, Han Li, Hui Liu, Yang Hou, Xu Dai, Ting Liu, Shiqi Jin

**Affiliations:** 1 Department of Radiology, The First Hospital of China Medical University, Shenyang 110001, China; 2 Department of Radiology, Shengjing Hospital of China Medical University, Shenyang 110001, China

**Keywords:** cardiac magnetic resonance, feature tracking, myocardial infarction, left atrial dysfunction

## Abstract

Background: LA function has been recognized as a significant prognostic marker in many cardiovascular diseases. Cardiovascular magnetic resonance feature tracking (CMR-FT) represents a promising technique for left atrial function evaluation. The size and location of myocardial infarction are important factors in the cause of adverse left ventricular remodeling, but the effect on the left atriam is unclear. Purpose: to investigate the effect of location and size of previous myocardial infarction (MI) on LA function using CMR-FT. Study type: retrospective. Population: patients formerly diagnosed with anterior MI (*n* = 42) or non-anterior MI (*n* = 40) and healthy controls (*n* = 47). Field Strength/Sequence: a 3.0T MR, Steady state free precession (SSFP), Phase-sensitive inversion recovery (PSIR). Assessment: infarct location and size were assigned and quantified by late-gadolinium enhancement (LGE) imaging. LA performance was analyzed using CMR-FT in 2- and 4-chamber cine images, including LA reservoir, conduit and booster pump function. Statistics: descriptive statistics, ANOVA with post Bonferroni correction, Kruskal–Wallis H, Spearman’s correlation, intraclass correlation coefficient. Results: Anterior MI patients had impaired LA reservoir function (LATEF, εs, SRs), conduit function (LAPEF, εe, SRs) and booster pump function (LAAEF, εa) compared with controls (*p* < 0.05). Non-anterior MI patients had impaired LA strain (εs, εe, εa; *p* < 0.05) but preserved LAEFs (*p* > 0.05). After adjusting the area of MI, there was no significant difference in the LA morphology and function between the anterior and non-anterior wall groups. Stratification analysis by MI size revealed that LA volumes and LAEFs were unchanged in patients with MI size ≤ 15% compared with controls (*p* > 0.05); only εs and εe were decreased (*p* < 0.05). Increased LAVIpre-a, LAVImin and decreased LATEF, and LAAEF were found in patients with MI size > 15% compared with the MI size ≤ 15% group (*p* < 0.05). LVSVI, εs and MI size were significant correlated with LAVI pre-a in multiple stepwise regression analysis. Data conclusions: The location of myocardial infarction is not a major factor affecting the morphology and function of the left atrium. Patients with MI size > 15% experience more pronounced post-infarction LA remodeling and dysfunction than MI size ≤ 15% patients.

## 1. Introduction

Left ventricular systolic and diastolic function is impaired after acute myocardial infarction (MI) and left atrial (LA) function also undergo changes. Impaired LA function is related to myocardial fibrosis in the left ventricular infarction and diastolic filling transferring stress from the left ventricle (LV) to LA [1]. The left atrium regulates left ventricular filling and maintains normal heart volume through the LA reservoir, conduit and booster pump function [2,3,4].

Percutaneous coronary intervention (PCI) has improved MI patient outcomes, but some patients still suffer adverse cardiovascular events, such as arrhythmias and heart failure, resulting in poor prognosis [5]. During the period of recovery from myocardial infarction after PCI, the infarct area gradually decreases, producing a degree of LA and LV recovery. Necrotic tissue begins to be absorbed after 1–2 weeks of acute MI and fibrosis occurs, forming a scar within 6–8 weeks. The final infarct size after healing of the injury represents replacement fibrosis [6]. Previous studies have shown more pronounced adverse LV remodeling and poorer prognosis in patients suffering acute anterior MI than non-anterior MI [7,8]. The process of LV remodeling in patients with myocardial infarction is very variable, partly depending on the infarct area. However, the left atrial myocardium is also supplied by the coronary arteries. Coronary occlusion or stenosis may affect atrial function, further worsening ventricular diastolic function. Taking into account the interaction of the left atrium and the left ventricle, we suspect that MI location and size (i.e., final infarct size after injury healing) are also major factors influencing the shape and function of the left atrium, but uncertainties remain. Some studies have indicated that an LA performance parameter such as LA volume may predict Major Adverse Cardiovascular Events (MACE) post-acute MI [9,10,11,12] and LA strain analysis may reveal subtle functional changes in the atrium before the LA maximum volume index increases [13,14]. Therefore, an accurate and comprehensive evaluation of left atrial morphology and function is expected to be instructive regarding future treatments in post-MI convalescent patients. Cardiovascular magnetic resonance (CMR) allows the size of acute and previous MI to be accurately determined by the late-gadolinium enhancement (LGE) technique. CMR-FT is a quantitative post-processing technique that tracks myocardial movement based on steady state free precession (SSFP) cine sequences [15,16]. It can comprehensively evaluate LA morphology and function, including LA volume, LA emptying fraction (EF), LA strain and strain rate. Therefore, the current analysis uses CMR-FT to evaluate the effect of location and final size of infarcts on impaired LA function in post-MI convalescent patients after PCI.

## 2. Methods

### 2.1. Patient Population

**Figure 1:**
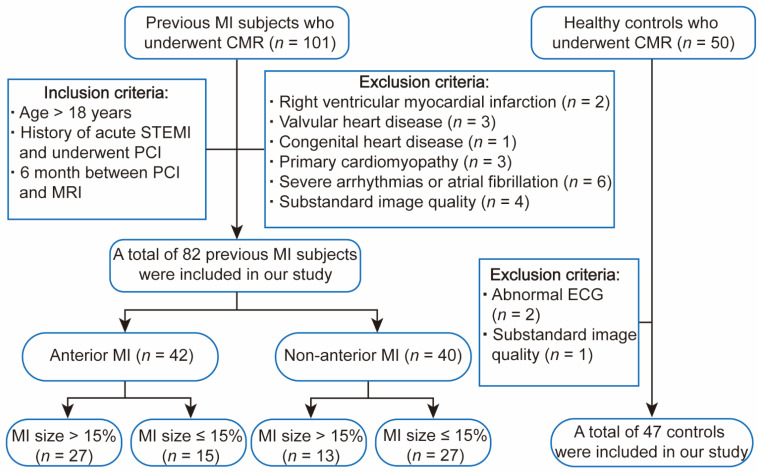
Subject flowchart. MI = myocardial infarction, CMR = cardiac magnetic resonance, ECG = electrocardiogram, STEMI = ST-segment elevation myocardial infarction, PCI = percutaneous coronary intervention.

A total of 101 consecutive patients who had previously suffered MI and received cardiac magnetic resonance imaging (MRI) between April 2017 and June 2021 were retrospectively enrolled. MI was diagnosed according to the criteria of The 2018 Fourth Universal Definition of Myocardial Infarction [17]. The inclusion criteria were as follows: (1) age > 18 years; (2) history of acute ST-segment elevation MI (STEMI); (3) 6 months between PCI and MRI. A total of 50 healthy volunteers with normal electrocardiogram (ECG) results, blood biochemical examination and cardiac function were recruited. Exclusion criteria for both patients and controls were as follows: right ventricular MI; valvular heart disease; congenital heart disease; primary cardiomyopathy; severe arrhythmias; atrial fibrillation or substandard image quality. Finally, 82 patients and 47 healthy controls were enrolled in this study. The patient recruitment procedure is shown in Figure 1. This study was approved by the committee of our hospital and written informed consents were obtained from all the participants.

### 2.2. CMR Imaging

Scans were performed on a 3.0-T MRI (MAGNETOM Verio, Siemens Healthcare, Erlangen, Germany) and retrospective cardiac gating was used. Balanced SSFP sequence with breath-hold generated cine CMR images comprising a stack of contiguous parallel short-axis slices covering the entire LV from base to apex plus three LV long-axis slice (2-, 3- and 4-chamber views). Slice thickness/spacings were 5 mm/1 mm for the long axis and 8 mm/2 mm for the short axis, repetition time (TR)/echo time (TE) was 3.8/1.3 ms and temporal resolution = 35 m. LGE imaging covered the entire heart: TR/TE = 750/1.6 ms, flip angle = 12°, bandwidth = 465 Hz/pixel and phase-sensitive inversion recovery (PSIR) occurred about 10 min after gadolinium-diethylenetriamine penta-acetic acid (Gd-DTPA) injection. The total acquisition time ranged from 30 to 40 min.

### 2.3. CMR Image Post-Processing

CMR image analysis was conducted by two radiologists with more than 3 years’ experience in cardiovascular imaging. The first observer (Z.H.) performed LA analysis while the second (JSQ) performed LV analysis. MRI examinations with reduced image quality or with suboptimal tracking were reviewed by a third experienced observer (T.L., a cardiologist with 8 years of experience in MRI-FT) and a consensus decision was reached. All the MR images were processed by CVI 4.2 (version 5.13.5, Circle Cardiovascular Imaging, Calgary, BC, Canada) over a time period of 20 min per case.

LV cardiac function and volume, including the volume of papillary muscles, were calculated from short-axis cine images [18]. LA volume and function were calculated from 4- and 2-chamber long-axis views. LA volume was estimated at LA end-diastole (LAVmax), LA end-early systole (LAVpre-a) and end-late LA late-systole (LAVmin). LA volume index (*LAVI*) was calculated by dividing LA volume by body surface area (BSA). The total left atrial (*LATEF*), passive atrial (*LAPEF*) and active atrial (*LAAEF*) emptying fractions were calculated according to the following formula:

$$LATEF=\frac{LAVmax-LAVmin}{LAVmax}\times 100$$

$$LAPEF=\frac{LAVmax-LAVpac}{LAVmax}\times 100$$

$$LAAEF=\frac{LAVpac-LAVmin}{LAVpre-a}\times 100$$

LA strain analysis was performed by a feature-tracking algorithm, and the borders of LA were manually delineated, excluding pulmonary veins, and then the curve of strain and strain rate changing with cardiac cycle were generated automatically by CMR-FTmodule of CVI 4.2 [19]. All contours were visually evaluated for adequate tracking and revised as necessary and LA total strain (εs, LA reservoir function), LA passive strain (εe, LA conduit function) and LA active strain (εa, LA booster pump function) were determined. Three SR parameters were evaluated, including reservoir strain rate (SRs, LA reservoir function), passive strain rate (SRe, LA conduit function) and active strain rate (SRa, LA booster pump function).

LGE cardiac MRI is best used to detect replacement fibrosis (i.e., final infarct size after injury healing) [6]. The location of the hyperintense myocardium with mean signal intensity (SI) >5 SDs greater than the reference region of interest on LGE post-contrast imaging indicated infarct location [18]. Infarction was defined as anterior when at least one of the basal anteroseptal, mid-anterior, mid-anteroseptal or apical anterior segments was involved [20]. Infarct size was estimated as the percentage of total LV volume and using the standard American Heart Association 17-segment model [21]. Measurements from the 17th segment were excluded from the final analysis to discount the partial volume effects at the apical cap. Infarct transmurality was determined as the percentage extent of the infarct along 100 equally spaced chords on each slice and calculated by dividing LGE area by the total area of the corresponding myocardial wall [22]. To obtain intra- and interobserver reproducibility data in LA strain and strain rate, both observers repeated analyses in 40 study participants (20 each from the MI and controls) after 2 months.

### 2.4. Statistical Analysis

Statistical analyses were performed by SPSS software (IBM SPSS Statistics 24.0, Armonk, NY, USA). Continuous variables were first tested for normality using Shapiro–Wilk’s test. Data are presented as percentage, means ± standard deviation (SD) or median (interquartile range, IQR), as appropriate. For normally distributed variables, Student’s *t* test was performed for comparisons of two groups and Chi^2^ and Fischer’s exact tests for proportions. One-way analysis of variance (ANOVA) with Bonferroni test was used for comparisons of multiple groups. Non-normally distributed variables were compared using the Mann–Whitney U test (comparisons of two groups) or Kruskal–Wallis H test (comparisons of multiple groups).

Intra- and inter-observer agreements of LA strain and strain rate were evaluated by intraclass correlation coefficient (ICC) and mean differences. Intraclass correlation coefficients (ICC) were assessed to evaluate intra- and inter-observer reproducibility as follows: ICC > 0.75: excellent; ICC = 0.60~0.74: good; ICC = 0.40~0.59: fair and ICC < 0.4: poor [23].

Pearson’s correlation analyses were used for continuous variables after verification of normality. The correlation coefficient, r, was interpreted as follows: 0.00 to 0.10: negligible correlation; 0.10 to 0.39: weak correlation; 0.40 to 0.69: moderate correlation: 0.70 to 0.90: good correlation and 0.90 to 1: strong correlation [13]. Multivariate linear regression was performed to associate LAVIpre-a with related parameters to establish the optimal regression equation.

All tests were two-tailed and a *p* value < 0.05 was considered statistically significant.

## 3. Results

### 3.1. Study Population

A total of 82 patients with previous myocardial infarction, consisting of 73 males (89%) and 9 females (11%) with mean age of 60 ± 10 years (range:32–80), were enrolled. Patients were assigned to anterior MI (*n* = 42, 51%) or non-anterior MI (*n* = 40, 49%) groups based on the location of LGE on post-contrast imaging. Controls were 47 gender-matched asymptomatic healthy participants with a similar age range (38 men, 9 women; mean age 60 ± 10 years). There was no significant difference in gender, BMI or BSA among the three groups (*p* > 0.05). No difference in N-terminal pro-B-type natriuretic peptide (NT-proBNP) between anterior MI and non-anterior MI patients was detected (*p* > 0.05). Demographic and clinical characteristics of participants are shown in Table 1.

### 3.2. Infarct Characteristics, Left Ventricular Volumes and Function

LVEF, Mitral annular plane systolic excursion (MAPSE)-inferior and MAPSE-anterior were significantly decreased in the anterior MI and non-anterior MI groups compared with controls (*p* < 0.001) and LVESVI, LVSVI and LVMI were increased (*p* < 0.001, Table 2). LVEF (43 [32–54] vs. 52 [37–62]) and MAPSE-anterior (9 [5–10] vs. 11 [6–14]) were lower in the anterior MI group compared with non-anterior MI (*p* < 0.05). LVEDVI was elevated in anterior MI patients relative to controls (85 [63–106] vs. 70 [56–114], *p* < 0.05) but no difference was found for non-anterior MI patients. Infarct size and transmurality were both significantly increased in the anterior MI group compared with the non-anterior MI group (17 [12–21] vs. 10 [6–19], *p* < 0.01; 45 [34–54] vs. 35 [28–43], *p* < 0.05).

### 3.3. CMR-FT Assessment of LA Volumetric and Functional Parameters

**Figure 2:**
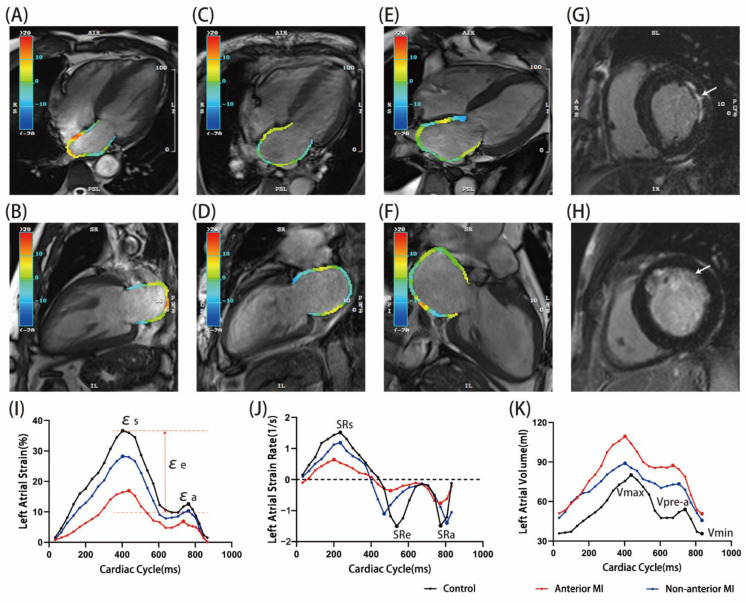
Typical examples of control ((**A**,**B**) and black line), non-anterior MI ((**C**,**D**,**G**) and blue line) and anterior MI ((**E**,**F**,**H**) and red line). (**A**,**C**,**E**) 4-chamber view of LA systolic phase, (**B**,**D**,**F**) 2-chamber view of LA systolic phase (the LA color varies strain parameters). (**G**) = lateral MI, (**H**) = anterior MI. (**I**–**K**) Curves showing LA strain, strain rate and volume in 25 phases, respectively. MI size was larger in the patient with anterior MI (16.9 vs.10.1%). εs = total strain; εe = passive strain; εa = active strain; SRs = total strain rate; SRe = passive strain rate; SRa = active strain rate.

Associations between MI location and LA parameters, including conventional atrial geometry and function and LA reservoir, conduit and booster pump function, are outlined in Table 3. Typical examples of LA volume and function are shown in Figure 2.

#### 3.3.1. LA Volumetric Parameters

**Figure 3:**
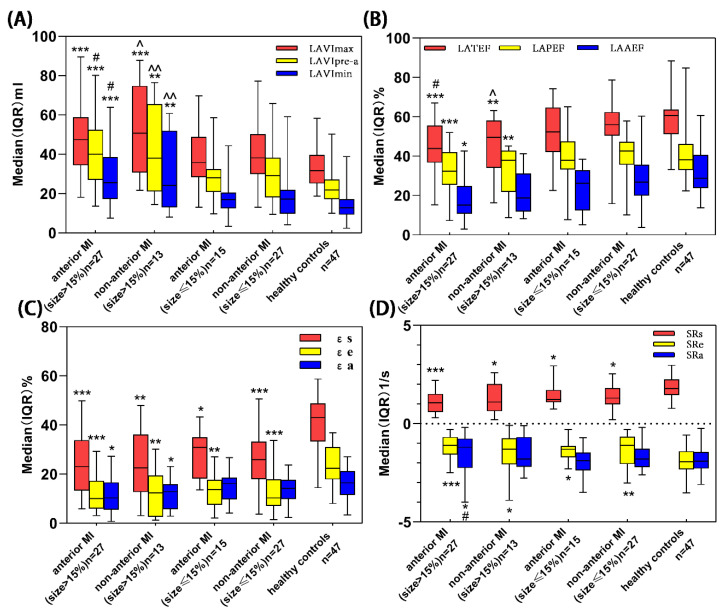
Kruskal–Wallis H was performed for comparisons of LA volume (**A**), LAEF (**B**), LA strain (**C**) and their corresponding strain rate (**D**) parameters among five groups (anterior MI (size > 15%), non-anterior MI (size > 15%), anterior MI (size ≤ 15%), non-anterior MI (size ≤ 15%) and healthy controls). 15% = median of MI size [8]. * *p* < 0.05 vs. healthy controls; ** *p* < 0.01 vs. healthy controls; *** *p* < 0.001 vs. healthy controls; ^ *p* < 0.05 vs. non-anterior MI (size ≤ 15%); ^^ *p* < 0.01 vs. non-anterior MI (size ≤ 15%); # *p* < 0.05 vs. anterior MI (size ≤ 15%). LA = left atrial, MI = myocardial infarction.

LA geometry indices, including LAVImax, LAVIpre-a and LAVImin, were all higher in both anterior MI and non-anterior MI patients compared with healthy controls (all *p* < 0.05). However, after adjusting MI size, there were no differences in LAVImax, LAVIpre-a and LAVImin between anterior MI and non-anterior MI patients (*p* > 0.05, Table 4). Further stratification of MI size showed that, compared with the controls, LA volumes were increased in both the anterior and non-anterior groups when MI size was >15% but no differences were found when MI size was ≤15% (Figure 3). In the anterior MI group, patients in the MI size > 15% subgroup had larger LAVIpre-a and LAVImin than in the MI size ≤ 15% subgroup. In the non-anterior MI group, patients in the MI size > 15% subgroup had larger LAVImax, LAVIpre-a and LAVImin than in the MI size ≤ 15% subgroup.

#### 3.3.2. LA Functional Parameters

Compared with the controls, significant impairments of LA reservoir (LATEF, εs, SRs), conduit function (LAPEF, εe, SRe) and booster pump function (LAAEF, εa) were found in anterior MI patients (all *p* < 0.05). However, impairments of only LA strain and strain rate (εs, εe, εa, SRs and SRe) were found in the non-anterior MI group with LATEF, LAPEF and LAAEF being well preserved. After adjusting MI size, there were no differences in LA functions between anterior MI and non-anterior MI patients (*p* > 0.05, Table 4). Further stratification of MI size showed that compared with controls and non-anterior MI (MI size ≤ 15%) patients, LATEF, LAPEF and LAAEF were significant impairments in anterior MI (MI size > 15%) group. All εs and εe values were impaired in all four size-stratified MI groups and εa was reduced in anterior and non-anterior MI groups with MI size > 15% (*p* < 0.05; Figure 3). In anterior MI group, patients with MI size > 15% subgroup had lower LATEF than in the MI size ≤15% subgroup. In non-anterior MI group, patients with MI size > 15% subgroup had lower LATEF and LAAEF than in the MI size ≤ 15% subgroup.

Consider that left atriam is supplied by LCX, the results showed that there were no significant differences in left atrial morphology and function between the LCX group and the non-LCX group (Table S1). After adjusting MI size, culprit coronary arteries are not a major factor affecting the morphology and function of the left atrium in convalescent MI patients (Table S2).

### 3.4. Correlation between MI Size and LA Functions

**Figure 4:**
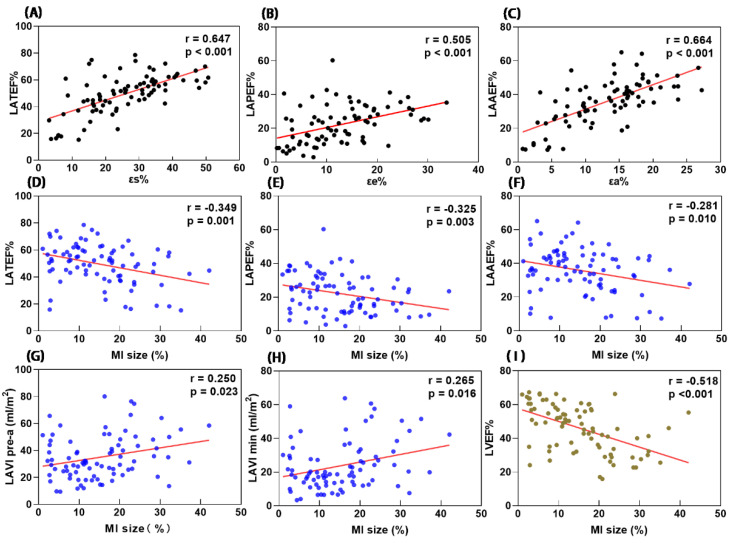
Scatter plots showing correlations of LATEF and εs (**A**), LAPEF and εe (**B**), LAAEF and εa (**C**), LA function and MI size (**D**–**F**), LA volume and MI size (**G**,**H**), LVEF and MI size (**I**). LA = left atrial, LV = left ventricular, EF = emptying fraction, εs = total strain, εe = passive strain, εa = active strain.

There were positive correlations between MI size and LAVIpre-a, LAVImin (r = 0.250, *p* < 0.05 and r = 0.265, *p* < 0.05; Figure 4). There were negative correlations between MI size and LA reservoir function (r[LATEF] = −0.349, *p* < 0.01), LA conduit function (r[LAPEF] = −0.325, *p* < 0.01) and booster pump function (r[LAAEF] = −0.255, *p* < 0.05; Table 5). The correlations between εs and LATEF (r = 0.647, *p* < 0.001), between εe and LAPEF (r = 0.505, *p* < 0.001) and between εa and LAAEF (r = 0.664, *p* < 0.001) were all moderate (Figure 4). The work undertaken by the left atrium depends on the volume at which it begins to contract, LAVIpre-a. LVSVI, MI size and εs were significantly associated with LAVIpre-a in multivariate linear regression (Table 6).

### 3.5. Reproducibility

The ICCs of the CMR-FT-derived LA strain and strain rate parameters were summarized in Table 7. As shown, intra-observer and inter-observer ICC coefficients were within the ranges of 0.89–0.97 and 0.77–0.96, respectively.

## 4. Discussion

The current study used CMR-FT to assess LA performance in patients with previous MI who had undergone PCI. The main findings were: (1) After adjusting the area of MI, there was no significant difference in the LA morphology and function between the anterior and non-anterior wall groups. These indicated that the location of myocardial infarction is not a major factor affecting the morphology and function of the left atrium. Patients with MI size > 15% experienced more pronounced post-infarction LA remodeling and dysfunction than patients with MI size ≤ 15%. (2) Furthermore, LA booster pump function was impaired, which may represent late stage left ventricular dysfunction and heart failure in the left atrium. LVSVI, εs and MI size were significantly associated with LA contractile function itself in post-MI patients.

The repair of myocardial infarction can be divided into three intersecting periods: inflammation, proliferation and maturation. The degree of post-myocardial infarction LV remodeling depends on infarct size and quality of repair [24]. An increasing number of recent studies have shown that the left atrium plays an important role in the maintenance of the entire cardiac cycle and cardiac function. Left atrial function is closely related to changes in overall heart function, which has significance for clinical research. Echocardiographic imaging of myocardial deformation can reveal impairments of LA function but CMR is the “gold standard” for the evaluation of cardiac morphology and function with high accuracy and repeatability that allows comprehensive evaluation of left atrial structure and function from multiple perspectives [5,12].

In terms of LA function, the reservoir and conduit functions contribute the most during early diastole while the booster pump function is the basis for LV filling during late diastole [13]. Therefore, quantification of LA function is helpful for disease staging and identification of patients at risk of adverse clinical events. The current results showed varying degrees of LA injury damage among patients with prior MI. Anterior MI patients had impaired LA reservoir (LATEF, εs, SRs), conduit (LAPEF, εe, SRe) and booster pump function (LAAEF, εa). In non-anterior MI patients, only εs, εe and εa decreased. LATEF, LAPEF and LAAEF were relatively preserved. These may represent a compensatory mechanism to maintain stroke volume and LV filling during mild diastolic dysfunction in accordance with the Frank-Starling law [25]. Previous studies have shown that, in acute MI, MI size, rather than location, may be an independent predictor of post-infarction prognosis [8,26]. Our study showed that, after adjusting the area of MI, there was no significant difference in the LA morphology and function between the anterior and non-anterior wall groups. These indicated that the location of myocardial infarction is not a major factor affecting the morphology and function of the left atrium. Stratification analysis during the current study revealed that no matter anterior or non-anterior MI, LA function in MI size >15% group is worse than in MI size ≤15% group. We think anterior MI patients experienced more severe LA injury than non-anterior MI patients due to the presence of a larger area of myocardial infarction. MI size was significantly correlated with LATEF, LAPEF and LAAEF. LAAEF, εa in patients with an MI of size >15% was decreased compared with controls whereas that of patients with MI size ≤15% was unchanged. Impairment of LA booster pump function (LAAEF, εa) may reflect reduced LA compliance with LV fibrosis in a stage of “decompensation”. It is likely that a late stage of left ventricular dysfunction is present and heart failure may be occurring the left atrium [27,28,29].

In terms of LA volume, left atrial size has been considered a biomarker of cardiovascular disease and a risk factor for heart failure, atrial fibrillation, myocardial infarction, stroke and sudden death [30]. The current study demonstrated the increased volume of the LA regardless of the site of MI compared with the controls. However, no significant differences in LA volumes were found between the controls and the subgroup with MI size of less than 15%. LAVIpre-a and LAVImin in the MI size > 15% group were larger than in the MI size ≤ 15% group, which demonstrated more severe diastolic dysfunction (DD). MI size was significantly correlated with LAVIpre-a, LAVImin. Reduced εs, εe and reserved LA volumes in the MI size ≤ 15% subgroup are consistent with previous studies demonstrating that LA performance, manifested as decrements in LA strain, is likely to be already impaired, due to increased LV pressure, before the LA begins to enlarge. In a study of 329 patients with diastolic dysfunction, 23% had impaired LA strains but normal LA geometry [31]. LA remodeling is a known characteristic of diastolic dysfunction but LA enlargement represents a cumulative effect of elevated LV filling pressure over time. Thus, LA strain may be more useful for detecting diastolic alterations before LA enlargement. Left atrial booster pump dysfunction may represent an advanced stage of the disease, and previous studies have shown that the work conducted by the LA depends on the volume at which it begins to contract (i.e., LAVIpre-a) [32,33]; we found that LVSVI, εs and MI size were significant correlated with LAVI pre-a in multiple stepwise regression analysis.

Furthermore, LA strain and strain rate were analyzed from 2- and 4-chamber cine images, including εs, εe, εa and their corresponding strain rates (SRs, SRe, SRa). These parameters have been employed and validated in previous LA CMR-FT studies [31,34]. The intra- and inter-observer reproducibility was good to excellent for all in both the current and in previous studies. Furthermore, the majority of LA deformation parameters were moderately associated with LAEFs, suggesting potential correlation between LA wall deformation and LA size.

## 5. Limitations

We acknowledge several limitations to the current study. Firstly, this is a retrospective single-center study with a relatively small number of patients. Strict inclusion and exclusion criteria were necessary to minimize the effect of confounding factors on LA function, which limited the sample size. Secondly, CMR-FT faces several challenges due to the variable anatomical shape of the left atrium, and differences in strain measurements caused by different CMR-FT models cannot be excluded. Standardization of postprocessing methods would be desirable to facilitate comparative analysis of LA deformation and reduce inter-model variability. Thirdly, little data relating to the impact of changes in LA function on cardiovascular events and long-term survival were available, and a substantial follow-up period would allow further validation of the predictive value of LA function in patients with previous MI.

## 6. Conclusions

During the recovery period from myocardial infarction after PCI, the location of myocardial infarction is not a major factor affecting the morphology and function of the left atrium. Detected by CMR-FT-derived LA parameters, patients with MI size >15% experience more pronounced post-infarction LA remodeling and dysfunction than MI size ≤15% patients.

## Figures and Tables

**Table 1:** Baseline characteristics.

Variables	Previous MI			
	Anterior MI (*n* = 42)	Non-Anterior MI (*n* = 40)	Controls (*n* = 47)	*p*-Value #
Age (years)	57.6 ± 13.2	59.4 ± 9.6	59.5 ± 10.2	0.762
Sex (male, *n*%)	37 (88)	36 (80)	38 (81)	0.783
Height (cm)	169.9 ± 5.9	171.0 ± 5.9	171.7 ± 6.9	0.790
Weight (kg)	68.1 ± 11.6	71.5 ± 11.9	70.2 ± 5.4	0.364
BMI (kg/m^2^)	24.5 ± 3.2	24.4 ± 3.4	24.1 ± 1.4	0.159
BSA (m^2^)	1.76 ± 0.2	1.81 ± 0.2	1.79 ± 0.1	0.281
Heart rate	68.2 ± 13.1	70.3 ± 13.5	71.4 ± 8.5	0.089
NT-proBNP (pg/mL)	485 (220–1304)	352 (161–930)	-	0.157
Characteristics culprit coronary artery *n* (%)				
Left anterior descending (*n*%)	42 (51)	-	-	
Left circumflex (*n*%)	-	13 (16)	-	
Right coronary artery (*n*%)	-	27 (33)	-	

Data were presented as mean ± standard deviations or percentages; ^#^ Significance of difference among three groups; BMI = body-mass index, BSA = body surface area, NT-proBNP = N-terminal pro-B-type natriuretic peptide.

**Table 2:** LV conventional parameters and infarct characteristics.

Variables	Anterior MI (*n* = 42)	Non-Anterior MI (*n* = 40)	Controls (*n* = 47)	*p*-Value #
LVEF (%)	43 (32–54) ***†	52 (37–62) ***	63 (59–67)	<0.001
LVEDVI (mL/m^2^)	85 (63–106) *	73 (59–96)	70 (56–114)	0.050
LVESVI (mL/m^2^)	44 (27–68) ***	32 (21–65) **	25 (19–31)	<0.001
LVSVI (mL/m^2^)	36 (29–40) ***	37 (29–43) **	45 (37–50)	<0.001
LVMI (kg/m^2^)	73 (60–86) *	74 (58–89) *	49 (43–58)	<0.001
MAPSE-inferior (mm)	10 (7–13) ***	12 (8–15) ***	16 (14–17)	<0.001
MAPSE-anterior (mm)	9 (5–10) ***†	11 (6–14) *	12 (11–14)	<0.001
MI size (%)	17 (12–21)	10 (6–19)	-	0.007
Transmurality (%)	45 (34–54)	35 (28–43)	-	0.013

Data were presented as median and interquartile range (IQR) deviations; # Significance of difference among three groups; * *p* < 0.05, compared with healthy controls; ** *p* < 0.01, compared with healthy controls; *** *p* < 0.001, compared with healthy controls; † *p* < 0.05, compared with non-anterior MI group; LV = left ventricle, EF = ejection fraction, EDVI = end-diastolic volume index, ESVI = end-systolic volume index, SVI = stroke volume index, LVMI = left ventricular mass index, MAPSE = mitral annular plane systolic excursion.

**Table 3:** LA volumetric and deformation parameters assessed by CMR-FT.

Variables	Anterior MI (*n* = 42)	Non-Anterior MI (*n* = 40)	Controls (*n* = 47)	*p*-Value #
Left atrial parameters				
LAVI max(ml/m^2^)	44.5 ± 16.1 ***	43.6 ± 18.5 **	32.5 ± 9.1	<0.001
LAVI pre-a (ml/m^2^)	36.5 ± 15.2 ***	33.6 ± 17.4 **	22.4 ± 7.9	<0.001
LAVI min(ml/m^2^)	25.4 ± 14.1 ***	22.0 ± 14.9 **	13.5 ± 6.5	<0.001
LA reservoir function				
LATEF (%)	46.0 ± 15.2 ***#	53.3 ± 13.5	59.9 ± 11.2	<0.001
εs (%)	25.6 ± 11.9 ***	25.9 ± 12.1 ***	40.2 ± 10.7	<0.001
LASRs (s^−1^)	1.3 ± 0.6 ***	1.3 ± 0.6 ***	1.9 ± 0.6	<0.001
LA conduit function				
LAPEF (%)	19.2 ± 9.8 ***#	25.3 ± 11.9	31.2 ± 11.1	<0.001
εe (%)	12.7 ± 7.4 ***	12.8 ± 8.3 ***	23.9 ± 7.8	<0.001
LASRe (s^−1^)	−1.2 ± 0.6 ***	−1.4 ± 0.9 **	−1.9 ± 0.7	<0.001
LA booster pump function				
LAAEF (%)	33.7 ± 14.3 *	38.1 ± 11.6	41.5 ± 13.3	0.024
εa (%)	12.9 ± 6.6 *	13.1 ± 5.8 *	16.3 ± 5.5	0.013
LASRa (s^−1^)	−1.7 ± 0.95	−1.6 ± 0.7	−1.9 ± 0.6	0.204

Data were presented as mean ± standard deviations; # Significance of difference among three groups; * *p* < 0.05, compared with healthy controls; ** *p* < 0.01, compared with healthy controls; *** *p* < 0.001, compared with healthy controls; # *p* < 0.05, compared with non-anterior MI group. LA = left atrial, VImax = maximal volume index, VIpre-a = pre-atrial contractile volume index, VImin = minimal volume index, TEF = total emptying fraction, PEF = passive emptying fraction, AEF = active emptying fraction, εs = total strain, εe = passive strain, εa = active strain. SRs = reservoir strain rate, SRe = passive strain rate, SRa = active strain rate.

**Table 4:** LA parameters of the anterior MI group compared with non-anterior MI group adjusting MI size.

	Anterior MI (*n* = 42)	Non-Anterior MI (*n* = 40)	Mean Difference (95% CI) *	*p* Value ^a^
LAVI max(mL/m^2^)	43.4 ± 2.7	44.8 ± 2.8	−1.34 (−9.167, 6.487)	0.734
LAVIpre-a (mL/m^2^)	35.2 ± 2.5	34.9 ± 2.6	0.337 (−6.963, 7.638)	0.927
LAVI min(mL/m^2^)	24.2 ± 2.2	23.3 ± 2.3	0.934 (−5.541, 7.409)	0.775
LATEF (%)	47.3 ± 2.2	51.9 ± 2.2	−4.684 (−11.041, 1.672)	0.146
εs (%)	26.1 ± 1.9	25.4 ± 1.9	0.726(−4.782, 6.235)	0.794
LASRs (s^−1^)	1.27 ± 0.1	1.29 ± 0.1	−0.021 (−0.301, 0.258)	0.880
LAPEF (%)	20.0 ± 1.7	24.5 ± 1.7	−4.471 (−9.372, 0.431)	0.073
εe (%)	12.7 ± 1.2	12.7 ± 1.3	0.29 (−3.61,3.67)	0.987
LASRe (s^−1^)	−1.26 ± 0.12	−1.34 ± 0.12	0.077 (−0.265, 0.419)	0.655
LAAEF (%)	34.7 ± 2.0	37.2 ± 2.1	−2.502 (−8.371, 3.367)	0.399
εa (%)	13.4 ± 0.96	12.7 ± 0.99	0.63 (−2.171, 3.431)	0.655
LASRa (s^−1^)	−1.67 ± 0.13	−1.60 ± 0.14	−0.064 (−0.451, 0.323)	0.742

Data were estimated marginal mean ± standard error; ^a^ One-way ANCOVA adjusted for MI size. * Mean difference = mean of the anterior MI subgroup—mean of the non-anterior MI subgroup. LA = left atrial, VImax = maximal volume index, VIpre-a = pre-atrial contractile volume index, VImin = minimal volume index, TEF = total emptying fraction, PEF = passive emptying fraction, AEF = active emptying fraction, εs = total strain, εe = passive strain, εa = active strain. SRs = reservoir strain rate, SRe = passive strain rate, SRa = active strain rate.

**Table 5:** Correlations of CMR-FT derived parameters with MI size in all patients with previous MI.

Parameters	MI Size (%)	
	*r*	*p*
LAVI max (mL/m^2^)	0.163	0.142
LAVI pre-a (mL/m^2^)	0.250	**0.023**
LAVI min (mL/m^2^)	0.265	**0.016**
LATEF (%)	−0.349	**0.001**
LAPEF (%)	−0.325	**0.003**
LAAEF (%)	−0.255	**0.021**
εs (%)	−0.136	0.225
εe (%)	−0.051	0.648
εa (%)	−0.195	0.078
LASRs (s^−1^)	−0.162	0.146
LASRe (s^−1^)	0.121	0.278
LASRa (s^−1^)	0.092	0.411

Pearson analysis was performed to investigate the correlations. Bold values indicate statistical significance. CMR = cardiovascular magnetic resonance, FT = feature tracking, LA = left atrial, MI = myocardial infarction, VI = volume index, EF = emptying fraction. εs = total strain, εe = passive strain, εa = active strain, SRs = total strain rate, SRe = passive strain rate, SRa = active strain rate.

**Table 6:** Univariate and multivariate regression analysis (backward stepwise) of association with LAVI pre-a (R^2^ = 0.445).

	Univariate Linear Regression			Multivariable Linear Regression		
	*β*	95% CI	*p* Values	*β*	95% CI	*p* Values
Age (years)	0.001	−0.522, 0.541	0.990			
Ln (NT-proBNP)	0.164	−0.370, 7.464	0.142			
LVEF (%)	−0.267	−1.065, −0.111	**0.015**			
LVEDVI (mL/m^2^)	0.443	0.153, 0.611	**<0.001**			
LVESVI (mL/m^2^)	0.405	0.180, 0.543	**<0.001**			
LVSVI (mL/m^2^)	0.258	0.131, 1.429	**0.019**	0.259	0.283, 1.279	**0.003**
LVMI (kg/m^2^)	−0.019	−0.157,0.152	0.865			
MI size (%)	0.264	0.149, 1.549	**0.016**	0.175	0.028, 1.057	**0.039**
Transmurality (%)	0.270	−0.153, 0.918	0.122			
MI location	−0.024	−14.164,12.352	0.832			
Heart rate (bpm)	0.214	−0.388, 1.099	0.053			
BMI (kg/m^2^)	0.133	−0.347, 3.096	0.234			
εs (%)	−0.603	−1.907, −1.040	**<0.001**	−0.584	−1.833, −1.020	**<0.001**
εe (%)	−0.473	−2.499, −1.035	**<0.001**			
εa (%)	−0.559	−3.520, −1.773	**<0.001**			

LV = left ventricular, EF = ejection fraction, MI location = anterior MI or non-anterior MI, LVMI = left ventricular mass index, VI = volume index, BMI = the body-mass index, εs = total strain, εe = passive strain, εa = active strain, β = standardized coefficient.

**Table 7:** Reproducibility of the LA function analysis by CMR-FT.

	Intra-Observer		Inter-Observer	
	ICC	CI	ICC	CI
εs (%)	0.974	0.952–0.986	0.963	0.931–0.980
εe (%)	0.937	0.884–0.966	0.858	0.748–0.922
εa (%)	0.951	0.909–0.974	0.880	0.785–0.935
LASRs (s^−1^)	0.969	0.943–0.984	0.853	0.738–0.919
LASRe (s^−1^)	0.886	0.796–0.938	0.774	0.612–0.874
LASRa (s^−1^)	0.951	0.910–0.974	0.807	0.664–0.893

ICC = intraclass correlation coefficient, CI = confidence interval, εs = total strain, εe = passive strain, εa = active strain, SRs = total strain rate, SRe = passive strain rate, SRa = active strain rate.

## Supplementary Materials

The following supporting information can be downloaded at: https://www.mdpi.com/article/10.3390/jcm11236938/s1, Table S1: LA volumetric and deformation parameters in LCX subgroup and non-LCX subgroup. Table S2: LA volumetric and deformation parameters of different culprit coronary arteries.
