# Supplementary material for: Effect of Infarct Location and Size on Left Atrial Function: A Cardiovascular Magnetic Resonance Feature Tracking Study
Source: J Clin Med. 2022 Nov 24;11(23):6938. doi: 10.3390/jcm11236938 (PMC9739184; doi:10.3390/jcm11236938)
Supplement: Supplementary file 1 [file jcm-11-06938-s001.zip › jcm-2034202-supplementary.pdf]

Table S1 LA volumetric and deformation parameters in LCX subgroup and non-LCX subgroup.

	**Non-LCX (n=69)**	**LCX (n=13)**	**P value**
LAVI max(ml/m^2)	43.0±17.1	49.7±17.3	0.204
LAVIpre-a (ml/m^2)	34.4±16.2	38.7±16.9	0.376
LAVI min(ml/m^2)	23.4±14.5	25.7±15.3	0.596
LATEF (%)	49.3±14.5	51.4±16.4	0.635
εs (%)	26.4±11.9	22.4±11.9	0.271
LASRs (s^{-1})	1.29±0.59	1.25±0.69	0.834
LAPEF (%)	21.9±10.2	23.9±16.3	0.559
εe (%)	12.9±7.5	11.2±9.6	0.453
LASRe (s^{-1})	-1.34±0.72	-1.06±0.79	0.203
LAAEF (%)	35.6±13.5	37.2±11.9	0.700
εa (%)	13.4±6.3	11.2±4.9	0.232
LASRa (s^{-1})	-1.68±0.86	-1.42±0.66	0.318

LA = left atrial, Vimax = maximal volume index, VIpre-a = pre-atrial contractile volume index, Vimin = minimal volume index, TEF = total emptying fraction, PEF = passive emptying fraction, AEF = active emptying fraction, εs = total strain, εe = passive strain, εa = active strain. SRs = reservoir strain rate, Sre = passive strain rate, Sra = active strain rate.

Table S2 LA volumetric and deformation parameters of different culprit coronary arteries

	**LAD (n=42)**	**RCA (n=27)**	**LCX (n=13)**	**P value[a]**	**P value[b]**
LAVI max(ml/m^2)	43.4±2.7	41.7±3.3	51.2±4.7	0.303	0.244
LAVI pre-a (ml/m^2)	35.2±2.5	32.2±3.1	40.5±4.4	0.278	0.302

LAVI min(ml/m^2)	24.2±2.2	21.3±2.7	27.4±2.2	0.316	0.428
LATEF (%)	47.3±2.2	53.1±2.7	49.6±3.9	0.069	0.267
εs (%)	26.1±1.9	27.1±2.3	21.7±3.3	0.443	0.397
LASRs (s^{-1})	1.27±0.1	1.33±0.1	1.22±0.2	0.762	0.844
LAPEF (%)	20.0±1.7	25.3±2.1*	22.8±3.0	**0.043**	0.162
εe (%)	12.7±1.2	13.5±1.5	11.1±2.2	0.683	0.680
LASRe (s^{-1})	-1.26±0.1	-1.48±0.1	-1.03±0.2	0.157	0.172
LAAEF (%)	34.7±2.0	37.8±2.5	35.9±3.6	0.305	0.640
εa (%)	13.4±0.96	13.7±1.2	10.6±1.7	0.373	0.286
LASRa (s^{-1})	-1.67±0.1	-1.70±0.2	-1.39±0.2	0.566	0.519

*$p < 0.05$ compared with LAD subgroup.

[a] One-way ANOVA with Bonferroni test compared among three groups. p values less than 0.05 are marked in bold.; [b] One-way ANCOVA adjusted for MI size.

LA = left atrial, Vimax = maximal volume index, VIpre-a = pre-atrial contractile volume index, Vimin = minimal volume index, TEF = total emptying fraction, PEF = passive emptying fraction, AEF = active emptying fraction, εs = total strain, εe = passive strain, εa = active strain. SRs = reservoir strain rate, Sre = passive strain rate, Sra = active strain rate.
